# Pennsylvania Hospital

**Published:** 1838-04-11

**Authors:** 


### CLINICAL LECTURES.

### PENNSYLVANIA HOSPITAL.

#### STRICTURE OF THE URETHRA.

*Wednesday, January* 31st, Dr. Harris commenced as follows:

I propose, gentlemen, to offer a few remarks on the subject of Strictures of the Urethra; and will afterwards introduce several patients affected with this distressing disease. They are all under treatment, and advancing towards a cure.

In order to understand accurately the diseases of the urethra, its length, calibre, and anatomical structure should be thoroughly examined. Discordant estimates have been formed of the length of this canal. Whately, who measured with precision forty-eight cases, fixes the extreme length to nine inches and six lines; and the shortest, to seven inches and nine lines. An African examined by Lisfranc, measured eleven inches. It has been long known indeed, that the genital organs of male and female Africans are more developed than those of the Caucasians.

Ducamp, Meckel, Home, and others, make the average length about eight inches.

We should also acquire data with regard to the diameter of the canal. We can never cure stricture, until the urethra is fully dilated to its natural size.

The estimates of Home, Lisfranc, and Phillips regarding the calibre of the canal, vary considerably. Phillips is of opinion that this difference arises from the different force used in propelling the waxen injection into the canal.

Amussat denies the existence of the dilatation at the fossa navicularis, and states that the urethra represents a cone, of which the base is represented posteriorly. The more common opinion is, however, that it is slightly enlarged through the prostate, and the commencement of the membranous portion, and contracts as it approaches the bulb,—enlarging again suddenly at the commencement of the spongy portion, and afterwards gradually diminishing to the meatus.

The urethra has been divided by anatomists into three portions. That succeeding to, and continuous with the neck of the bladder, has been named the prostatic portion—next is the membranous portion, and the third or remaining portion extends to the termination of the glans. Some writers have named also a bulbous portion; a distinction entirely unnecessary.

The prostatic portion commences at the neck of the bladder, and terminates at the anterior part of the prostate gland. Its length, according to Ducamp, who appears to be the most accurate observer, is from twelve to fifteen lines.

The second and narrower part of the urethra, which succeeds to the prostatic, is named the membranous portion. Its length, according to the same writer, is ten lines.

The next or terminating portion of the urethra, has been named the spongy, from its being surrounded by erectile tissue. Its length is dependent on the greater or less development of the organ. This portion of the canal commences at an enlargement called the bulb, and terminates at another enlargement called the glans penis. From the circumstances already stated, its length is variable, but is generally about seven or eight inches.

Two views have been entertained with regard to the anatomical structure and physiology of the urethra—the one supporting, and the other denying the muscularity of the canal. Mr. Hunter believed that "the substance of the urethra is muscular, and is therefore capable of contracting the canal so much as to close it up entirely. This makes it subject to diseases peculiar to muscle in general; *which is indeed the only proof* we have of its being muscular."

Mr. Hunter gave no anatomical facts in proof of the muscularity of the urethra; and though the same opinions have been urged by Sir Everard Home, we must decline receiving their bare assumptions for truths, particularly as such views are entirely at variance with the best ancient, as well as modern anatomists.

It may be therefore stated with some confidence, that a spasmodic stricture does not exist, because the tissue of the urethra is not capable of taking on a spasmodic action. Stricture then, has been well defined by Sœmmering to be a thickening of the mucous and cellular membranes of the urethra, to a greater or less extent, produced by inflammation. Sir Charles Bell entertains the same opinion. Lisfranc describes stricture as that state of the urethra in which the canal cannot resume its ordinary capacity, in consequence of the existence of a pathological condition.

If the urethra were originally muscular and contractile, the thickening and callosity of the part must be attended with loss of such contractile power.

Surgeons have confounded the action of the muscles surrounding the urethra, with the powers of the urethra itself. In order to settle this point, Bell instituted some well contrived experiments. He introduced an ivory ball into the urethra, to which a thread was attached, when the canal was found to possess no power to force it forwards. When the ball was covered with irritating ointment, the same want of contractility was apparent. The experiment was made on that part of the urethra anterior to the ejaculator seminis. When, however, the ivory instrument was pushed within the grasp of this muscle, a little effort was found sufficient to throw it forward. Other experiments were made which tended to establish the same point, but which a want of time will not enable me to detail.

It is easy to account for the spasms in the posterior part of the urethra, since five inches of that part of the canal are surrounded by muscles.

The sensibility of the urethra governs the contraction of these muscles. A strict attention to their action in the perineum will explain many of the phenomena attending what is improperly called spasmodic stricture. The sudden diminution in the calibre of the urethra, may arise from a sudden tumefaction of the lining membrane of the urethra —from a strong fluxionary movement of blood into the corpus spongiosum, or from the powerful contraction of the perineal muscles.

The term spasmodic stricture, should therefore be abolished, as this affection can only consist of a permanent thickening, or change of structure in the diseased part of the urethra. The space occupied by this altered structure may be from a line in extent to four or five inches. There may be one contraction, or there may be several.

There is no part of the urethra entirely exempt from stricture; the whole being subject to inflammation, and to the altered structure which it causes. The prostatic portion of the canal is, however, less subject to this disease than other parts. I have never seen a case of stricture in this position, though I am aware that two cases of the kind are reported by Lallemand. The most frequent seat of this disease is from four to six inches from the mouth of the urethra. Phillips states that out of one hundred and seventy-three cases, one hundred and twenty-nine were located between the points last named.

If the question is asked, why the disease is most frequently located in this situation, it will be found difficult to answer. Acute urethritis which has succeeded to contagion, is generally at a point anterior to the curvature of the canal; but this disease commonly results from chronic phlogosis, which has a tendency to be propagated backwards, and to become stationary at the space to which I have alluded. It is the nature of this form of inflammation, as it diminishes in intensity and extent, to concentrate on a particular point; thus gaining in profundity what it loses in surface.

Obstructions to the flow of urine are attended with nearly the same symptoms. Patients do not often notice the first symptoms of stricture. For example, they will have a stricture of some extent without observing any remarkable diminution of the stream of urine. It does diminish in size, however, in proportion to the extent of the obstruction. As the disease further advances, the stream becomes forked or scattered, and is sometimes voided by drops. The stricture at first forms but slowly, but when it has so much increased that the urethra is not relaxed by the force of the urine, then the subsequent advances are more rapid. The patient now occupies more time in urining, and a smaller quantity is evacuated at each period. The desire to urine is felt more frequently; the patient is obliged to rise from bed several times in the night—the discharge of urine is accomplished by long efforts, and with more or less pain and tumefaction of the penis. After having passed a certain quantity, in a few minutes renewed efforts are made, and a further discharge is accomplished; a proof that the bladder was not emptied during the first effort. This is a very common symptom in protracted stricture.

The difficulty in emptying the bladder at one effort, arises from the great length of time required to accomplish this end. In cases of stricture, the expulsion of urine is not complete, because the time which is necessary for the evacuation must exceed that during which the bladder can maintain contraction.

In this stage of the disease exposure to cold, acid drinks, indulgence in venery and stimulating drinks will often place the patient in imminent danger. The passage of urine is so difficult, that instead of a jet to some distance, it falls vertically between the feet. Frequently, indeed, it escapes by drops, and will require ten minutes to pass three or four ounces. After it ceases to flow the desire to pass more is still pressing.

Under such circumstances the efforts to expel the urine are painfully distressing; the exertions of the patient are such as to cause a general tremor of the extremities, a livid flushing of the face, and perspiration to start from every pore.

I have seen cases where the bladder and its sphincter were so distended by the accumulated urine, that it escaped in a continued and involuntary manner, thus giving the appearance of incontinence.

The urine being thus confined, becomes irritating, and not unfrequently gives rise to inflammation. An afflux of blood is thrown into the vessels of the diseased part, occasioning swelling, by which the passage is closed, and retention of urine is the consequence.

Phlogosis of the urethra, as in other mucous tissues, is attended often with a secretion of a viscid substance which lodges at the stricture, and thus plugs up the aperture and prevents the passage of urine. This secretion often acquires considerable density, and has been forced out in a form which Racine has compared to a piece of vermicelli.

When the patient labours under retention of urine, the danger which menaces him is imminent. The bladder becomes hard, and distended sometimes, until it rises as high as the umbilicus; the desire to urine being most pressing and painful. The abdomen now becomes tender on pressure, the circulation is accelerated, followed by nervous tremors and delirium. If the patient thus situated is not speedily relieved, the bladder and all that portion of the urethra posterior to the stricture, inflames, becomes either ulcerated or gangrenous, and bursts either into the cavity of the peritoneum, or into the cellular texture around the rectum, perineum, or scrotum. In the former case the accident necessarily proves fatal, and in the others where the constitution is vigorous, sloughs may form and be thrown off, followed by urinal fistula. If, however, free incisions be speedily made around the rectum, perineum, and scrotum, so as to allow the extravasated urine to escape, the sloughing may be prevented.

Strictures are, I believe, always produced by inflammation. They may result from gonorrhœa, from the irritation of calculi lodged in the urethra, from contusions, or from irritating or acrid injections. Two years ago, I had a boy eight years of age, under my care, in this institution, affected with an unyielding stricture, produced by a severe blow in the perineum. The assertion, then, that strictures always arise from venereal affections is not correct. The same pathological condition may be found in the œsophagus, the intestines, and lacrymal passages, where gonorrhœal matter is never brought in contact.

A variety of treatment has been adopted by different surgeons in the management of strictures of the urethra. No one of them is adapted to all cases, and yet perhaps each may be found useful under some particular conditions. The several methods shall be briefly detailed. The instruments used for the purpose of removing this disease consist of bougies formed of waxed linen, of catgut, of gum elastic, of flexible metal—apparatus for applying caustic, and for cutting or dividing the stricture. Here are all these instruments spread on the table before you.

These bougies act by producing mechanical dilatation; and their internal pressure causes an absorption of the hardened and altered structure, which constitutes the disease.

If the common or waxed bougie is used, it should be of a large size, smeared with warm olive oil, and passed down the urethra to the obstructed point. If the surgeon is unable to pass the instrument, a smaller and smaller one is to be tried, until one is obtained which may be forced through. The operator may sometimes succeed by allowing the bougie to press on the stricture for a short period, and then push it forward.

There is sometimes a difficulty in ascertaining whether a small bougie has entered the stricture, or has been only bent. When the pressure is removed from the instrument, and it recoils, we may be certain that it has not entered the stricture.

When a bougie is withdrawn the point should be examined, and if it be flattened, or the waxen coat pushed up, we may be sure that it has entered the narrowed part of the canal to an extent corresponding with these impressions. If no great irritation follow the first introduction, the following day the instrument should be again used, and allowed to remain as long as the patient can endure it without suffering. If the introduction be roughly or forcibly made, the urethra may be torn, and hemorrhage to a considerable extent may be the consequence. Such is the great sensibility of the urethra of some patients, that even with the gentlest management, sickness, fainting, and chills may ensue.

It should be a rule not to introduce the bougie until the unpleasant effects of a previous introduction have subsided. While in some cases the instrument may be daily used, in other instances, such is the irritability of the urethra, that days must elapse before the operation can be advantageously repeated.

When the sensibility of the urethra is very great, the use of bougies should be suspended for a time, and leeches should be applied to the perineum, and the patient confined to bed. Under such circumstances the warm bath will be found useful.

When the morbid sensibility of the urethra is removed, the instrument must be resumed, and continued until one of the largest size is easily introduced.

In cases where the stricture has nearly closed the urethra, I have found the common or waxed bougie entirely inefficient. It is too flexible and yielding when small to enable the operator to apply any force to the obstructed point.

In the worst cases of strictures I have found the elastic gum catheters and bougies more useful. Even a small instrument constructed of this material, may be strengthened by a strong iron wire passed within it, so that the operator may apply some degree of force.

When, however, the hard and unyielding stricture is located in the membranous part of the urethra, even this instrument will fail to penetrate the obstruction. Even when strengthened by the wire, it yields at the convex part, and little force is exerted on the disease. To obviate this difficulty I contrived an instrument, which has proved eminently successful in many cases. It consists of a strong and full sized silver catheter open at the extremities, with a series of silver probes of various sizes. In order to prevent the edges of the catheter from wounding the urethra, the end is bevelled in, so that the aperture is in the centre of the instrument. The catheter is now passed down to the stricture, and held firmly by an assistant in this situation, while the surgeon passes down the smallest probe against the obstruction. The probe being supported by the sides of the catheter, any necessary degree of force may be used. The probe passing through its sheath in the centre of the urethra, no fears can be apprehended of making a false passage, if proper care be observed. When it has passed, others of larger size can be made to follow. Instead of the silver probe I have used the catgut in the same manner. The advantage of this substance is, that when it once enters the stricture, moisture is absorbed by it, which causes the instrument to swell to twice its original magnitude. After the stricture is dilated by such means to some extent, then the dilitation must be completed by the ordinary bougies.

I have seen this simple instrument prove successful in so many instances, that I feel great confidence in recommending it to the notice of the profession. It may be purchased of Mr. Rorer, a surgical instrument maker in North Sixth street, beyond Market. Bougies made of gum elastic, or of flexible metal, may now be used until the urethra is dilated to its natural magnitude. After the disease has been entirely overcome, there is frequently a liability to return, unless proper precautions are observed. These consist in an occasional introduction of the bougie at intervals of a few weeks for a year. This is so important, that the surgeon should always instruct the patient in the use of this instrument himself.

When large bougies are required, I prefer the flexible metallic to all others. They are smooth and unirritating, have sufficient firmness for introduction, and may be used without injury for a long period. The gum elastic bougie or catheter, becomes rough and irritating after a few introductions. It has been objected to on account of liability to break, but in a practice of more than twenty years I have never witnessed an accident of this kind.

There are two other methods of destroying stricture; the one chemical, the other mechanical. The first, is the application of a caustic substance, which destroys the animal solid constituting the disease; the second, is the division of the stricture by means of a cutting instrument introduced into the canal.

Caustic was used in such cases by Ambrose Paré, Loyseau, and Wiseman. Hunter adopted a similar treatment, but instead of applying lunar caustic through a canula, as was the practice of his predecessors, he used a common wax bougie, at the extreme end of which was deposited the escharotic. An aperture was made in the end of the instrument in which was placed the caustic, and around which the wax linen was pressed so as to cover it, except at one point. After having the bougie thus armed, it is then requisite to ascertain its precise location. With this view, an unarmed wax bougie is introduced down to the obstruction, and you then measure the exact distance from the stricture to the mouth of the urethra. The distance being marked on the armed bougie, it is well oiled, and passed rapidly down to the stricture against which it is pressed for about a minute. The operation should not be repeated in less than two or three days, and should be thus continued, until the disease is destroyed. The best rule perhaps is not to repeat a second application, until the eschar caused by the first, has been detached.

Unquestionably caustic will cause the destruction of strictures, yet there are objections to its use of a serious character. With the greatest care the sound portion of the urethra is often materially injured. When the slough is cast off, the canal is excoriated, producing ardor urinæ, a profuse, purulent discharge, chordee, swelled testicle, and sometimes suppression of urine. If you apply the caustic to the anterior part of the stricture, the lower, rather than the superior parietes of the urethra is most affected—the sound, rather than the diseased structure is most extensively destroyed, and false passages thus become no uncommon accident.

Many of the French and some of the English surgeons prefer the vegetable to the lunar caustic as an escharotic. The most common rule in its application is to arm the *porte-caustic* and pass it through a canula, by which the urethra is protected. This instrument enters the stricture and applies the caustic to the sides of it, and not to the anterior part as Hunter and Home recommended. The advantages of the vegetable over the lunar caustic are that it is more perfectly under our control. The free application of olive oil, will at once neutralise the vegetable caustic by converting it into soap.

One of the arguments in favour of caustic is that it destroys the indurated substance in the urethra, and thus renders the patient less liable to the disease. This is a mistake. The disease as often returns after it has been treated by caustic, as by the dilating bougie. After both methods, the occasional use of the bougie, and temperate living, is necessary to complete protection.

With regard then to the use of caustic my opinion is that it is more or less dangerous to apply it to the anterior part of a stricture, because it is as liable to burn the sound as the diseased portion; and that there is no necessity of applying it to the side of the disease, because, if you can introduce a *porte-caustic*, you can as readily pass a bougie of the same size. When this instrument has been once forced through a stricture, we may fairly consider it under our control. If a small bougie has once entered the obstruction, there will be little difficulty in passing a larger one, and so on, until the urethra is dilated to its natural size. I therefore believe that where the urethra is not entirely obliterated the disease may be cured by the dilating instruments already mentioned, and without resorting to the harsher treatment by escharotics.

[Dr. Harris now introduced three patients labouring under strictures of the urethra. They were in various stages of treatment. He explained the proper manner of introducing bougies and then passed them into the bladder. One of the patients, whose urethra was irritable, was thrown into a chill by the operation.]

